# The association between cardiovascular health and cognition in adults with Down syndrome

**DOI:** 10.1186/s11689-023-09510-z

**Published:** 2023-12-06

**Authors:** Lauren Frank, Brian Helsel, Danica Dodd, Amy E. Bodde, Jessica C. Danon, Joseph R. Sherman, Daniel E. Forsha, Amanda Szabo-Reed, Richard A. Washburn, Joseph E. Donnelly, Lauren T. Ptomey

**Affiliations:** 1 School of Medicine, The University of Kansas Medical Center, 3901 Rainbow Boulevard, Kansas City, KS 66160 USA; 2 Department of Internal Medicine, University of Kansas Medical Center, 3901 Rainbow Boulevard, Mail Stop 1073, Kansas City, KS 66160 USA; 3 Department of Neurology, The University of Kansas Medical Center, 3901 Rainbow Boulevard, Kansas City, KS 66160 USA; 4 Ward Family Heart Center, Children’s Mercy Kansas City, Kansas City, MO USA

**Keywords:** Down syndrome, Fitness, Blood pressure, Physical activity, Exercise, Cognition, Alzheimer’s disease, Dementia, Intellectual disabilities

## Abstract

**Introduction:**

Evidence in the general population suggests that predictors of cardiovascular health such as moderate to vigorous physical activity (MVPA), cardiorespiratory fitness, and systolic blood pressure are associated with cognitive function. Studies supporting these associations in adults with Down syndrome (DS) are limited. The purpose of this study was to examine the associations between systolic blood pressure, cardiorespiratory fitness, and MVPA on cognition in adults with DS.

**Methods:**

This is a cross-sectional analysis using baseline data from a trial in adults with DS. Participants attended a laboratory visit where resting blood pressure, cardiorespiratory fitness (VO_2 Peak_), and cognitive function (CANTAB® DS Battery) were obtained. The cognitive battery included tests measuring multitasking, episodic memory, and reaction time. Physical activity (accelerometer) was collected over the week following the laboratory visit. Pearson correlations and linear regressions were used to measure the impact of systolic blood pressure, cardiorespiratory fitness, and MVPA on cognitive outcomes.

**Results:**

Complete data was available for 72 adults with DS (26.8 ± 9.3 years of age, 57% female). At baseline, VO_2 Peak_ (21.1 ± 4.2 ml/kg/min) and MVPA were low (14.4 ± 14.4 min/day), and systolic blood pressure was 118.3 ± 13.3 mmHg. VO_2 Peak_ was correlated with simple movement time (rho =  − 0.28, *p* = 0.03) but was not significant using a linear regression controlling for age and sex. Systolic blood pressure was significantly associated with episodic memory (first attempt memory score: *β* =  − 0.11, *p* = 0.002; total errors: *β* = 0.58, *p* = 0.001) and reaction time (five-choice movement time: *β* = 4.11, *p* = 0.03; simple movement time: *β* = 6.14, *p* = 0.005) using age- and sex-adjusted linear regressions. No associations were observed between MVPA and multitasking, episodic memory, or reaction time.

**Conclusion:**

Predictors of cardiovascular health, including cardiorespiratory fitness and systolic blood pressure, were associated with some aspects of cognition in adults with DS. While future research should examine the role of improved cardiovascular health on delaying decreases in cognitive function and dementia in adults with DS, we recommend that health care providers convey the importance of exercise and cardiovascular health to their patients with DS.

**Trial registration:**

NCT04048759, registered on August 7, 2019.

## Introduction

Down syndrome (DS) is a genetic condition caused by extra chromosome 21 material in all or some cells of the body and is the most common chromosomal abnormality associated with an intellectual disability [1]. The current incidence of DS is 1 in every 650 live births [2]. The life expectancy for individuals with DS has increased dramatically in recent decades from 4 years in the 1970s due to high rates of congenital heart defects, to 60 years currently [3].

However, with this increase in life expectancy comes an increased risk of Alzheimer’s disease (AD) and dementia. By the age of 65 years, the cumulative incidence of dementia exceeds 90% [4] and is the leading cause of death in individuals with DS [5]. Adults with DS also have an earlier onset of dementia with cognitive declines starting at ~ 40 years of age [4, 5]. Research in midlife adults without DS suggests that factors related to cardiovascular health such as increased moderate-to-vigorous physical activity (MVPA), cardiorespiratory fitness, and systolic blood pressure are associated with components of cognition including attention, memory, and executive function [6–8]. Additionally, MVPA [9] cardiorespiratory fitness [10] and systolic blood pressure [11] at midlife are associated with a lower incidence of later life dementia, possibly by increasing cognitive reserve so that a person can better maintain cognitive performance even with neuropathologic changes related to AD [12].

Previous research in adults with DS suggests that MVPA may be associated with cognitive function [13, 14]. However, we are unaware of previous studies that have examined the association of systolic blood pressure and cardiorespiratory fitness on cognition in adults with DS without dementia. Thus, this project aims to use baseline data collected from a physical activity trial in adults with DS to examine the association of systolic blood pressure, cardiorespiratory fitness, and MVPA on cognition in adults with DS.

## Methods

### Overview

This is a cross-sectional analysis using baseline data from a physical activity trial in adults with DS without dementia [15]. Participants attended a single laboratory visit for assessments of resting blood pressure, cardiorespiratory fitness, and cognition, then wore an accelerometer belt to assess MVPA for 7 days following the laboratory visit. All data were collected between January 2020 and November 2022. This study was approved by the University’s Institutional Review Board. Informed consent and assent were obtained from participants and their parent/legal guardians prior to data collection.

### Participants

Participants were adults (≥ 18 years of age) with DS who were living at home with a parent or guardian or in a supported living environment with a caregiver who agreed to serve as a study partner. Additional inclusion criteria were mild to moderate intellectual disabilities, sufficient functional ability to understand directions, ability to communicate through spoken language, ability to participate in physical activity, and walk 10 feet unassisted and having Internet access in the home. Participants were excluded if they had dementia as determined by the Dementia Screening Questionnaire for Individuals with Intellectual Disabilities (DSQIID) [16]; participated in a regular exercise program (i.e., ≥ 20 min/days ≥ 3 days/weeks); had a serious medical risk, such as cancer or a recent cardiac event as assessed by the primary health care provider; or were unable to participate in MVPA. Participants were recruited using flyers, list-serves, and social media posts by local organizations that provide services to adults with DS (e.g., Down syndrome societies, day service programs, and clinics and community developmental disability organizations funded by Medicare). Participants could be living with a family member, alone or with roommates, or in a supported group living environment. Caregivers of potential participants were asked to contact the study coordinator who answered questions about the study and administered the initial participant eligibility screener which included questions about the participants’ functional ability, communication skills, and the DSQIID. A home visit or video conference meeting was scheduled with those remaining interested and potentially eligible to determine final eligibility and to obtain consent.

### Outcomes

Resting blood pressure was measured on the non-dominant arm with participants seated in a chair and both feet flat on the ground. An aerobic step was placed under the feet of those participants who were unable to place their feet on the ground. After 5 min of quiet rest in a chair, blood pressure was taken in duplicate with 2 min separating the first and second measurements. A third measurement was taken for systolic and diastolic values ≥ 5 mmHg apart, and the average of the two closest measures was used for analysis. Systolic blood pressure represents the amount of pressure exerted by blood against your artery walls during ventricular contraction, whereas diastolic blood pressure is the amount of pressure exerted against your artery walls during ventricular rest between contractions. Only systolic blood pressure was used as an outcome since it is a better predictor of cardiovascular outcomes in midlife [17, 18].

Cardiorespiratory fitness (VO_2 Peak_) measured in ml/kg^/^min was assessed using a maximal treadmill test, following the protocol described by Fernhall et al. developed for adults with DS [19]. Expired O_2_ and CO_2_ were measured using indirect calorimetry (ParvoMedics TrueOne 2300, Salt Lake City, Utah), which was calibrated with known volume and gas concentrations prior to each test according to the manufacturer’s recommendations. Values were averaged over 15-s intervals across the treadmill protocol. The exercise test was terminated if participants were unable to maintain the treadmill speed or requested that the test be stopped at any time during the treadmill protocol. Otherwise, tests were terminated when participants achieved two or more of the following criteria: (1) volitional exhaustion; (2) a plateau in VO_2_, i.e., < 150 ml/min or heart rate (HR) < 2 beats/min with increased work rate; (3) a HR within 5 beats/min of HR_Peak_ predicted using the formula of Fernhall et al. [19]; and (4) a respiratory exchange ratio ≥ 1.0. Only participants who achieved a respiratory exchange ratio ≥ 1.0 or had a HR within 10 beats/min of their HR_Peak_ were included in the analysis.

### Physical activity

MVPA and sedentary time were assessed using an ActiGraph wGT3XBT tri-axial accelerometer (ActiGraph LLC, Pensacola, FL) worn on the non-dominant hip at the anterior axillary line during waking hours for 7 consecutive days. Vertical axis data aggregated over 60 s epochs from the ActiGraph devices were initialized and downloaded using Actilife software version 6.13.3 (ActiGraph LLC, Pensacola, FL). Non-wear time was defined as at least 90 consecutive minutes of zero counts, with an allowance for 1–2 min of movement between zero and 100 counts/min [20]. Counts ≥ 20,000·min^−1^ were considered spurious [21]. A wear time ≥ 8 h on at least 3 days including 1 weekend day was required for inclusion in the analysis. Vertical axis ActiGraph cut points used for adults in the 2003–2004 and 2005–2006 cycles of NHANES were used to classify minutes of sedentary time (< 1.0 MET, ≤ 100 counts/min) and MVPA (≥ 3 METs, ≥ 2020 counts/min) [22, 23].

Cognitive function was assessed using the Cambridge Neuropsychological Test Automated Battery for DS (CANTAB®, Cambridge Cognition, LTD, Cambridge, UK) [24, 25] administered on an iPad® in a quiet room. The CANTAB® DS Battery, which has been used in previous trials in individuals with DS [26, 27], includes measures of executive function, episodic memory, and processing speed. CANTAB® DS Battery measures have demonstrated sensitivity to disease-specific cognitive deficits in DS, including those related to hippocampal dysfunction, and sensitivity to changes in cognitive function associated with AD [28]. The following CANTAB tests were used:

#### Multitasking task

Multitasking task, a measurement of executive function, assessed the ability of participants to ignore task-irrelevant information. An arrow was presented on either side of the screen pointing in either direction (right or left). Participants had to pay attention to either the side of the screen where the arrow appears or the direction of the arrow (indicated by SIDE or DIRECTION on the screen), by pressing a button on the left or right corner of the screen, respectively. Four key outcomes were collected from the multitasking task: incongruency cost, which is the difference (in milliseconds) between the median latency of response (from stimulus appearance to button press) on the trials that were congruent versus the trials that were incongruent; reaction latency, which is the median latency of response (from stimulus appearance to button press); multitasking cost, which is the difference (seconds) between the median latency of response (from stimulus appearance to button press) during assessed blocks in which both rules are used versus assessed blocks in which only a single rule is used; and total incorrect, which is the number of trials for which the outcome was an incorrect response.

#### Episodic memory

The paired associates learning test assessed visual episodic memory. Participants were presented with boxes on the screen in which different visual patterns are shown one by one. After the encoding phase, the different patterns were shown in the middle of the screen and participants had to select the box in which the pattern was previously presented. Outcome measures included the first attempt memory score, which assessed the number of times a participant was able to correctly recall a pattern previously displayed on the computer screen on their first attempt with higher scores indicating better memory, and the total errors, which are the number of times the subject chose the incorrect box for a stimulus on assessment problems plus an adjustment for the estimated number of errors they would have made on any problems, attempts, and recalls they did not reach.

#### Reaction time

The reaction time task, a measure of attention and psychomotor speed, assessed reaction times for motor and mental responses. Previous research suggests that simple reaction time is correlated to memory and verbal fluency, and choice reaction time is related to language, orientation, and verbal fluency [29–31]. During the task, circles (one for the simple task and five for the choice mode) were shown at the top of the screen in which a random yellow light appeared. Participants had to hold a button on the bottom of the screen and release it to select the circle above in which they detected the yellow light as fast as possible and then return their fingers to the hold button. Outcome measures included simple and five-choice reaction time, which is the medium duration required for participants to release the response button following presentation of the target stimulus (milliseconds) calculated across trials when the stimulus could appear in only one location or in any one of five locations, respectively, and simple and five-choice movement time, which is the median time (milliseconds) taken for a subject to release the response button and select the target stimulus after it flashed yellow on screen, calculated across trials when the stimulus could appear in only one location or in any one of five locations.

### Statistical analysis

Sample characteristics were described as mean ± standard deviation or frequency (percentage) for demographic (i.e., age, race, ethnicity, level of support), anthropometric (i.e., height, weight, body mass index), systolic and diastolic blood pressure, and cardiorespiratory fitness (VO_2 Peak_) measures. Pearson correlations were used to describe the association between (1) systolic blood pressure, (2) cardiorespiratory fitness, and (3) MVPA with 10 cognition measures within 3 domains (i.e., multitasking cost, episodic memory, and reaction time). Linear regressions were used to further explore the impact of systolic blood pressure, cardiorespiratory fitness, and MVPA on cognition in adults with DS while adjusting for age, sex, and body mass index. R version 4.2.2 was used for this analysis [32].

## Results

Baseline data were collected from 82 adults with DS, of those 72 completed the full cognitive battery and were included in this analysis. Participants were 26.8 ± 9.3 years of age, 57% female, and ~ 75% were living with a parent. Sample characteristics stratified by sex are presented in Table 1. Pearson correlations between systolic blood pressure, cardiorespiratory fitness, and MVPA and cognitive outcomes are presented in Table 2, and the impact of systolic blood pressure, cardiorespiratory fitness, and MVPA on cognitive outcomes using linear regression is presented in Table 3.

**Table 1:** Sample characteristics of adults with Down syndrome included in the analysis

**Characteristic**	**Overall**, *N* = 72^a^	**Male**, *N* = 31^a^	**Female**, *N* = 41^a^
Age	26.8 ± 9.3	29.3 ± 10.8	24.8 ± 7.5
Race			
African American	4 (5.6%)	1 (3.2%)	3 (7.3%)
American Indian	1 (1.4%)	1 (3.2%)	0 (0%)
Asian	1 (1.4%)	1 (3.2%)	0 (0%)
Mixed race	4 (5.6%)	0 (0%)	4 (9.8%)
Pacific Islander	1 (1.4%)	1 (3.2%)	0 (0%)
White	61 (85%)	27 (87%)	34 (83%)
Ethnicity			
Hispanic	7 (9.7%)	3 (9.7%)	4 (9.8%)
Non-Hispanic	65 (90%)	28 (90%)	37 (90%)
Severity of intellectual disability			
Mild	53 (74%)	21 (68%)	32 (78%)
Moderate	19 (26%)	10 (32%)	9 (22%)
Height (cm)	151.7 ± 8.6	159.0 ± 5.5	146.1 ± 5.8
Weight (kg)	75.3 ± 17.7	79.2 ± 20.0	72.3 ± 15.3
Body mass index	32.7 ± 7.2	31.3 ± 7.8	33.8 ± 6.5
Systolic blood pressure	118.3 ± 13.3	122.8 ± 13.5	114.9 ± 12.2
Diastolic blood pressure	65.4 ± 8.7	67.6 ± 8.2	63.8 ± 8.8
MVPA (min/day)	14.4 ± 14.4	14.9 ± 12.7	14.1 ± 15.7
VO_2_ (ml/kg/min)	21.1 ± 4.2	23.0 ± 3.9	20.0 ± 4.1

^a^Mean ± SD; *n* (%)

**Table 2:** Pearson correlations between factors related to cardiovascular health and cognitive outcomes in adults with Down syndrome

	**Systolic blood pressure *N* = 63**		**VO**_**2**_** peak *N* = 61**		**MVPA *N* = 59**	
	rho	*p* value	rho	*p* value	rho	*p* value
Multitasking						
Incongruency cost	0.03	0.83	–0.11	0.42	0.07	0.58
Reaction latency	0.18	0.17	–0.18	0.16	0.04	0.74
Multitasking cost	–0.08	0.56	0.11	0.40	–0.04	0.78
Total incorrect	–0.14	0.27	–0.11	0.39	–0.10	0.45
Episodic memory						
First attempt memory score	–0.40	0.001	–0.06	0.67	0.04	0.78
Total errors	0.43	< 0.001	–0.01	0.95	0.01	0.96
Reaction time						
Five-choice movement time	0.29	0.02	–0.22	0.09	–0.01	0.96
Five-choice reaction time	0.09	0.51	–0.21	0.11	–0.06	0.63
Simple movement time	0.35	0.005	–0.28	0.03	–0.01	0.93
Simple reaction time	0.27	0.03	–0.13	0.32	–0.09	0.48

**Table 3:** Examining the association between factors related to cardiovascular health and cognition using linear regression

Outcome: cognition	Systolic blood pressure *N* = 63			VO_2 Peak_ *N* = 61			MVPA *N* = 59		
	*β*	SE	*p* value	*β*	SE	*p* value	*β*	SE	*p* value
Multitasking									
Incongruency cost	0.44	0.87	0.615	− 2.18	2.62	0.409	0.16	0.78	0.843
Reaction latency	1.97	1.79	0.274	− 6.08	5.19	0.246	1.92	1.61	0.238
Multitasking cost	− 0.96	1.23	0.436	4.29	3.84	0.270	− 0.28	1.32	0.835
Total incorrect	− 0.19	0.20	0.332	− 0.58	0.61	0.347	− 0.14	0.18	0.426
Memory									
First attempt memory score	− 0.11	0.03	0.002	− 0.08	0.12	0.517	− 0.01	0.04	0.806
Total errors	0.58	0.16	0.001	0.17	0.56	0.767	0.15	0.18	0.408
Reaction time									
Five-choice movement time	4.11	1.85	0.030	− 7.07	4.84	0.150	1.36	1.65	0.413
Five-choice reaction time	1.68	2.25	0.458	− 3.84	3.41	0.265	0.90	2.17	0.678
Simple movement time	6.14	2.09	0.005	− 10.10	5.30	0.062	1.85	1.88	0.331
Simple reaction time	4.12	2.13	0.058	− 3.85	5.00	0.444	0.46	1.94	0.815

*SE* Standard error, *BP* Blood pressure

Age- and sex-adjusted linear regressions

**Fig. 1:**
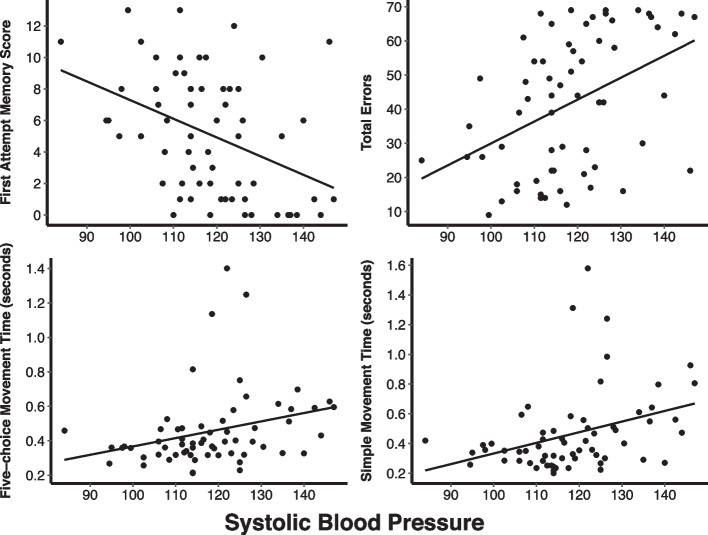
The linear association between systolic blood pressure (mmHg) and cognitive function variables in adults with Down syndrome

Resting blood pressure was collected from 63 participants. Mean systolic blood pressure was 118.3 ± 13.3 mmHg, and 1 participant met the criteria for hypertension (i.e., ≤ 140/90 mmHg). Systolic blood pressure was significantly correlated with both episodic memory measures (both *p* ≤ 0.001) and 3 measures of reaction time: five-choice movement time (rho = 0.29, *p* = 0.02), simple movement time (rho = 0.35, *p* = 0.005), and simple reaction time (rho = 0.27, *p* = 0.03). When controlling for age and sex, systolic blood pressure had a significant association with both episodic memory measures, five-choice movement time, and simple movement time (all *p* < 0.05, Fig. 1). No associations were observed with any of the multitasking measures.

### Cardiorespiratory fitness

**Fig. 2:**
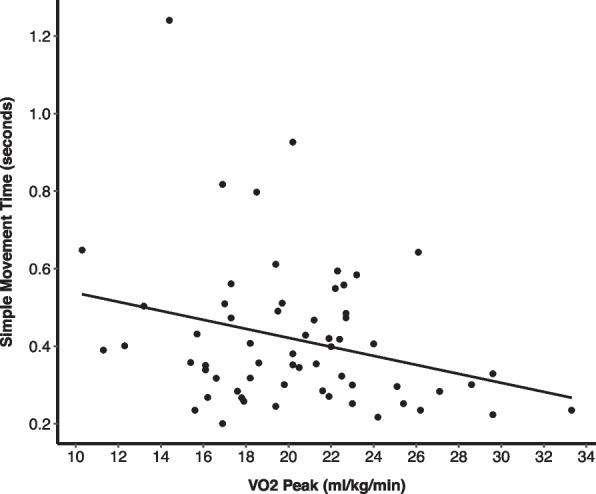
The linear association between cardiorespiratory fitness (VO_2peak_) and cognitive function in adults with Down syndrome

Valid cardiorespiratory fitness was obtained from 61 participants, and the mean VO_2 Peak_ was 21.1 ± 4.2 ml/kg/min. Cardiorespiratory fitness was significantly correlated with simple movement time (rho =  − 0.28, *p* = 0.03). There was no association between VO_2 Peak_ and cognitive outcomes after adjusting for age and sex in a linear regression; however, the crude association between VO_2 Peak_ and simple movement time was statistically significant (*β* =  − 11.61, *p* = 0.03, Fig. 2). No associations were observed with multitasking or episodic memory measures.

### Moderate to vigorous physical activity

Valid accelerometer data was obtained from 59 participants, and the mean MVPA was 14.4 ± 14.4 min/day. MVPA was not significantly correlated with any of the cognitive measures (all *p* > 0.05), nor were any associations observed when using linear regression controlling for age and sex.

## Discussion

The results of the current study observed an association between systolic blood pressure and episodic memory as well as 2 measures of reaction time. Additionally, we observed a correlation between cardiorespiratory fitness and simple movement time which measured psychomotor speed; however, when controlling for sex and age, this association was no longer significant. Additionally, the current study did not observe an association between MVPA and any of the cognitive measures.

Adults with DS have very low cardiorespiratory fitness [33, 34] and low normal blood pressure [35, 36]. For example, in the current study, cardiorespiratory fitness was ~ 21 ml/kg/min, compared to ~ 34–44 ml/kg/min observed in adults of similar age without DS [37]. Several factors have been proposed to explain the low cardiorespiratory fitness and blood pressure in individuals with DS, with the most likely cause being autonomic dysfunction [33, 34, 38]. Autonomic dysfunction includes lower maximal HR, resulting in lower cardiac output and exercise capacity [33, 39], and impaired blood pressure control and peripheral blood flow regulation, which potentially impairs the person with DS’s ability to direct blood flow to working muscles during exercise [33, 34, 38]. It is unclear how autonomic dysfunction may influence the association between cardiovascular health and cognition in adults with DS; however, the results of the current study suggest that blood pressure and cardiorespiratory fitness may still be associated with some aspects of cognition.

We are unaware of previous research examining the association between systolic blood pressure or cardiorespiratory fitness and cognitive function in adults with DS. In the non-DS population chronic high blood pressure, known as hypertension, is associated with cognitive decline and risk of later life dementia [11, 40]. For example, a recent systemic review and meta-analysis [11] found moderate-quality evidence indicating that midlife hypertension was related to a 1.19 to 1.55-fold excess risk of cognitive disorders. Dose–response analyses of 5 studies included in the review indicated that midlife systolic blood pressure of > 130 mm Hg was associated with a 34% increased risk of dementia. The mechanisms underlying hypertension-related cognitive changes are complex and not yet fully understood [17]. However, it is thought that hypertension causes oxidative stress and inflammation, which disrupts the blood–brain barrier and leads to white matter lesions [41]. White lesions are thought to affect cognition by impairing the connectivity between the anterior thalamus and the frontal cortex [41]. However, individuals with DS have lower blood pressure compared with age-matched controls [36], and it is believed that individuals with DS may be protected from hypertension [42, 43]. Thus, it was unknown if blood pressure would be associated with cognition in adults with DS. However, the results of the current study found a strong correlation between lower systolic blood pressure and better memory scores and reaction time scores, suggesting that systolic blood pressure may indeed play a role in cognitive function in this population.

The results of the current study found a correlation between cardiorespiratory fitness and simple movement time, although this relationship was no longer significant when controlling for age and sex. We are unaware of any previous research examining the association of cardiorespiratory fitness on cognition in adults with DS. In the non-DS population, cardiorespiratory fitness has been associated with improved reaction time in the general population [44] and in adults with multiple sclerosis [45]. In adults without DS, most research has focused on the relationship between increased cardiorespiratory fitness and episodic memory [46–48]. The results of the current study did not observe an association between cardiorespiratory fitness and episodic memory. However, previous work by our group in 27 adults with DS [49] observed improvements in episodic memory (*p* = 0.048) after 12 weeks of exercise [49], suggesting that exercise, and secondary increases in cardiorespiratory fitness, may be associated with improvements in episodic memory. Future research examining the impact of increased cardiorespiratory fitness on cognition is warranted.

MVPA is thought to impact cognition by multiple pathways, including improving other cardiovascular risk factors (e.g., hypertension, obesity, dyslipidemia) and improving cardiorespiratory fitness (VO_2 Peak_) [50]. We did not observe any association between MVPA and cognition, which contrasts with previous research in adults with DS suggesting that MVPA is closely associated with cognition [13, 14]. For example, Fleming et al. [13] reported that the percentage of time spent in MVPA was significantly correlated with 8/9 cognitive outcomes used in Alzheimer’s Biomarker Consortium DS (ABC-DS) cognitive battery [51]. Similarly, a study by Pape et al. [14] which followed 214 participants across 12 months observed that higher MVPA at baseline was associated with a 62% reduced risk of decline in memory and orientation at 12 months. Additionally, previous intervention studies in adults with DS have demonstrated the potential for acute or short-term (≤ 12 weeks) MVPA to improve cognitive outcomes [9, 49, 52–54]. Trials examining the impact of long-term increases on MVPA on cognition are warranted.

This study benefits from the use of device-based assessments of MVPA and the use of directly measured cardiorespiratory fitness (i.e., VO_2 Peak_), as well as a relatively large and diverse sample of adults with DS. However, it is limited as the data is cross-sectional and all participants were from a sample of adults with DS who had agreed to participate in a program focused on the promotion of physical activity. One major limitation of the current study is that all participants had low levels of cardiorespiratory fitness (~ 21 ml/kg/min) and MVPA (~ 14 min/day) which was required for inclusion in the clinical trial; therefore, there was a smaller range of MVPA and cardiorespiratory fitness in this sample compared with the general population of individuals with DS, limiting our ability to detect a correlation with cognition. Similarly, participants were younger and cognitively stable; thus, we were unable to examine the cognition of older adults more at risk of dementia or who had already progressed to Alzheimer’s disease.

## Conclusion

Cardiovascular risk factors, including low cardiorespiratory fitness and high systolic blood pressure, were associated with some aspects of cognition in adults with DS. While future research should examine the role of improved cardiovascular health on delaying decreases in cognitive function and dementia in adults with DS, we recommend that health care providers convey the importance of exercise and cardiovascular health to their patients with DS, especially given the high prevalence of dementia in adults with DS.

## Data Availability

Deidentified individual participant data (including data dictionaries) will be made available, in addition to study protocols, the statistical analysis plan, and the informed consent form. The data will be made available upon publication to researchers who provide a methodologically sound proposal for use in achieving the goals of the approved proposal. Proposals should be submitted to the *corresponding author at lptomey@kumc.edu.*

## Abbreviations

DS: Down syndrome
AD: Alzheimer’s disease
MVPA: Moderate to vigorous physical activity
DSQIID: Dementia Screening Questionnaire for Individuals with Intellectual Disabilities
